# Zscan4 Contributes to Telomere Maintenance in Telomerase-Deficient Late Generation Mouse ESCs and Human ALT Cancer Cells

**DOI:** 10.3390/cells11030456

**Published:** 2022-01-28

**Authors:** Jiameng Dan, Zhongcheng Zhou, Fang Wang, Hua Wang, Renpeng Guo, David L. Keefe, Lin Liu

**Affiliations:** 1 State Key Laboratory of Medicinal Chemical Biology, Department of Cell Biology and Genetics, College of Life Sciences, Nankai University, Tianjin 300071, China; loyal.zhongcheng@163.com (Z.Z.); wangh2@mskcc.org (H.W.); guorp@njau.edu.cn (R.G.); 2 State Key Laboratory of Primate Biomedical Research, Institute of Primate Translational Medicine, Kunming University of Science and Technology, Kunming, Yunnan 650500, China; Yunnan Key Laboratory of Primate Biomedical Research, Kunming, Yunnan 650500, China; 3 Department of Obstetrics and Gynecology, New York University Langone Medical Center, New York, NY 10016, USA; fang.wang@nyulangone.org (F.W.); david.keefe@nyulangone.org (D.L.K.)

**Keywords:** Zscan4, telomere, ES cells, ALT, DNA methylation, H3K9me3

## Abstract

Proper telomere length is essential for indefinite self-renewal of embryonic stem (ES) cells and cancer cells. Telomerase-deficient late generation mouse ES cells and human ALT cancer cells are able to propagate for numerous passages, suggesting telomerase-independent mechanisms responding for telomere maintenance. However, the underlying mechanisms ensuring the telomere length maintenance are unclear. Here, using late generation telomerase KO (G4 Terc^-/-^) ESCs as a model, we show that *Zscan4*, highly upregulated in G4 Terc^-/-^ ESCs, is responsible for the prolonged culture of these cells with stably short telomeres. Mechanistically, G4 Terc^-/-^ ESCs showed reduced levels of DNA methylation and H3K9me3 at Zscan4 promoter and subtelomeres, which relieved the expression of Zscan4. Similarly, human ZSCAN4 was also derepressed by reduced H3K9me3 at its promoter in ALT U2 OS cells, and depletion of ZSCAN4 significantly shortened telomeres. Our results define a similar conserved pathway contributing to the telomere maintenance in telomerase-deficient late generation mESCs and human ALT U2OS cancer cells.

## 1. Introduction

Mammalian telomeres consist of repetitive G-rich sequences and associated proteins at the ends of linear chromosomes and function in the maintenance of chromosomal stability and integrity [1,2]. Telomeres are primarily maintained by active telomerase which is composed of telomerase reverse transcriptase (TERT), telomerase RNA (TERC), and dyskerin [2]. Telomerase is expressed highly in a subset of stem cells, as well as in most immortal and cancer cells, presumably to support their indefinite proliferation and self-renewal [3,4]. Most mammalian somatic cell types do not express telomerase activity, such that telomeres shorten progressively with each cell cycle and the end replication problem ensues [5,6].

Previously, we have generated ES cell lines with high efficiency from wild type (WT, Terc^+/+^), heterozygous (Terc^+/-^), and early to late generation of Terc^-/-^ mouse blastocyst (G1, G3 and G4 telomerase RNA null blastocyst) [7]. Interestingly, late generation telomerase-deficient *mTerc^-/-^* mouse ES cells (G4 Terc^-/-^) show telomere shortening and dysfunction compared with WT ES cells, yet can sustain their proliferation for numerous passages without telomerase activity [7], suggesting the involvement of telomerase-independent alternative lengthening of telomeres (ALT) mechanisms responding for telomere maintenance. Similarly, about 10–15% of human cancer cells lack detectable telomerase activity, and many of these use an ALT mechanism to maintain telomeres in cancer growth (reviewed in [8]). However, the mechanisms ensuring telomere length maintenance in both cases remain elusive. Furthermore, whether late generation telomerase-deficient *mTerc^-/-^* mouse ES cells and ALT cancer cells share the same or similar mechanisms to maintain telomere homeostasis remains to be determined.

Telomeres and subtelomeres are enriched with repressive DNA and histone modification (e.g., DNA hypermethylation and H3K9me3/H4K20me3 histone modifications) that are important negative regulators of telomere length [9], forming an inhibitive effect to the genes nearby, named Telomere Position Effect (TPE) [10,11,12]. These DNA and histone modifications at subtelomeric regions were reduced in late generation Terc^-/-^ mouse embryonic fibroblast (MEF) cells [13].

*Zscan4*, a gene with unique expression pattern, expressed specifically in two-cell embryos and transiently in sporadic ES cells (1–5%) at any given time, marks a transient 2C-like state of mouse ES cells [14,15,16,17]. In 2C-like cells (2CLCs), *Zscan4* and many other 2C-specific genes are direct targets of *Dux*, which is necessary and sufficient for the transition of mouse ESCs to 2CLCs [18,19,20]. Functionally, Zscan4 is required for preimplantation embryonic development, lengthening telomeres promptly by activating telomere recombination and maintaining exceptional genomic stability [16,17,21]. *Zscan4* knockdown in mESCs leads to telomere shortening, abnormal karyotypes (chromosome deletion and fusion), proliferation defects, and, ultimately, apoptosis, while *Zscan4* re-expression could rescue those abnormalities [16]. *Zscan4* knockdown in early embryos delays the progression from two-cell to four-cell stage and produces blastocysts that fail to implant or proliferate in blastocyst outgrowth culture [17]. Most recently, TRF2 was shown to be dispensable for telomere protection in pluripotent stem cells through the upregulation of *Zscan4* [22]. In contrast, TRF2 is critically required for telomere protection in differentiated somatic cells. Depletion of *Zscan4* in TRF2-null ES cells results in re-appearance of telomere fusion that normally seen in TRF2-null differentiated somatic cells, while induced expression of *Zscan4* in TRF2-null differentiated somatic cells prevents telomere fusion [22], supporting the critical roles of Zscan4 in maintaining genomic stability in ES cells. Furthermore, Zscan4 was also found to specifically recognize a subset of (CA)_n_ microsatellites and protect mouse two-cell embryos from DNA damage, which ensures the successful progression of early embryo development [23]. Surprisingly, 2C-like mESCs were reported to be associated with global DNA hypomethylation [14,15]. Mechanistically, we previously reported that as mESCs proliferate, they sporadically enter a 2C-like totipotent state, resulting in a burst of Zscan4 expression [15]. Zscan4, via its ZNF4 motif, recruits the Uhrf1-Dnmt1 complex and, through self-association mediated by its SCAN domain, brings two or more Uhrf1-Dnmt1 heterodimers into proximity. This facilitates Uhrf1′s E3 ligase-mediated ubiquitination of Uhrf1 and Dnmt1, likely through cross actions between Uhrf1 molecules. Subsequent Uhrf1 and Dnmt1 degradation leads to global DNA demethylation, which facilitates telomere recombination and elongation [15]. This mechanism somehow explains Zscan4′s effects on promoting telomere recombination and elongation through chromatin de-condensation with reduced DNA methylation. However, the exact mechanisms on how Zscan4 promotes telomere recombination and elongation remain to be elucidated.

Interestingly, both mouse *Zscan4* and human ZSCNA4 genes locate at subtelomeric regions, with mouse *Zscan4* at short p arm of chromosome 7 while human ZSCAN4 at short p arm of chromosome 19, indicating that *Zscan4* might be repressed by TPE. Here, we show that reduced repressive epigenetic modifications at *Zscan4* promoters de-repress *Zscan4*, which contributes to telomere maintenance in both late generation Terc^-/-^ ES cells and telomerase-deficient human ALT-U2 OS cancer cells.

## 2. Materials and Methods

### 2.1. Cell Culture

Both WT and G4 Terc^-/-^ ES cell lines derived from C57BL/6J mice were isolated and characterized previously in our laboratory [7]. J1 ES cells were cultured without feeder. The ES cell culture medium consisted of knock-out DMEM (Gibco) with 15% FBS (Hyclone), 1000 U/mL mouse leukemia inhibitory factor (LIF) (ESGRO, Chemicon), 0.1 mM non-essential amino acids, 0.1 mM β-mercaptoethanol, 1 mM L-glutamine, and penicillin (100 U/mL), and streptomycin (100 µg/mL). For culture of all ES cell lines, the medium was changed daily, and cells were routinely passaged every two days. HeLa cells were cultured with high-glucose DMEM (Gibco) with 10% FBS (Hyclone), 0.1 mM non-essential amino acids, 1 mM L-glutamine, and penicillin (100 U/mL), and streptomycin (100 µg/mL). The ALT positive U2 OS cell medium was HeLa medium plus 0.2 mM non-essential amino acids.

### 2.2. Stable Zscan4 RNAi in G4 Terc^-/-^ ES Cells, ALT-U2 OS Cells, and ZSCAN4 KO in U2 OS Cells

For stable knockdown of mZscan4 in ES cells, control and mZscan4 shRNAs (Table S1) were cloned into pSIREN-RetroQ (Clontech) and introduced into Plat-E cells for packaging of retrovirus. G4 Terc^-/-^ ES cells were infected with indicated retrovirus and selected with 1.5 µg/mL puromycin for about 10 days. The resistant clones were picked and expanded for further culturing. For stable knockdown of human ZSCAN4 in U2 OS cells, initially, EGFP in RNAi lentivirus vector pLL3.7 was replaced with neomycin between NheI/EcoRI sites and named as pLL3.7-neo [24]. After initial test on RNAi efficiency using seven different shRNA sequences against human ZSCAN4, four hZscan4 shRNA and control sequences (Table S1) were cloned into pLL3.7-neo lentivirus vectors. The resultant vectors were introduced into 293 T cells with helper vectors to produce lentivirus, and 48 h later the indicated lentivirus were used to infect U2 OS cells and selected with 700 µg/mL G418 for 10 days. The pLL3.7-EGFP vector was used to monitor the infection efficiency. For ZSCAN4 KO in U2 OS cells, CRISPR-Cas9 technology was used to generate stable KO clones. Guide RNA (gRNA) targeting exon 3 of human ZSCAN4 gene was cloned into a targeting vector, and single clones were picked and expanded for further experiments.

ZSCAN4 gRNA sequence: ACAGCAATAATTCATATGCA.

### 2.3. Cell Cycle Analysis

Cells were fixed in freshly prepared 70% ethanol at 4 °C overnight, then centrifuged at 1000× *g* for 5 min and stained with propidium iodide (PI) at 37 °C for 30 min in water bath. FACS analysis was used to determine cell cycle progression.

### 2.4. Gene Expression Analysis by Quantitative Real-Time PCR 

Total RNA was isolated from cells using RNeasy mini kit (QIAGEN). Two micrograms of RNA were subject to cDNA synthesis using M-MLV Reverse Transcriptase (Invitrogen). Real-time quantitative PCR reactions were set up in duplicate with the FastStart Universal SYBR Green Master (ROX) (Roche, Hvidovre, Denmark) and run on the iCycler iQ5 2.0 Standard Edition Optical System (Bio-Rad) using primers. Most primers were designed using the IDT DNA website. The primers used are listed in Table S3. 

### 2.5. Immunoblot Analysis

Cells were washed twice in PBS, collected, lysed, and boiled in SDS sample buffer at 99 °C for 5 min; Equal amounts of total proteins of each cell extracts were resolved by 10–12% Bis-Tris SDS-PAGE and transferred to polyvinylidinedifluoride membranes (PVDF, Millipore, Burlington, MA, USA). Nonspecific binding was blocked by incubation in 5% skim milk in TBST at room temperature for 1–2 h. Blots were then probed with various primary antibodies, anti-Zscan4 (AB4340, Millipore, 1:1000), Dnmt1 (sc10221, Santa Cruz, 1:500), Dnmt3a (ab13888, Abcam, 1:1000), Dnmt3b (ab13604, Abcam, 1:1000), Tet2 (Kind gift from Dr. Jinsong Li from SIBS, 1:1000), H3K9me3 (ab8898, Abcam, 1:2000), H3 (ab1791, Abcam, 1:2000), and β-actin ((P30002, Abmart, 1:5000) by overnight incubation in 5% skim milk in TBST at 4 °C. Immunoreactive bands were then probed for 1–2 h at room temperature with the appropriate horseradish peroxidase-conjugated secondary anti-Rabbit IgG-HRP (GE Healthcare, NA934V, 1:5000), or anti-mouse IgG-HRP (Santa Cruz, sc-2031, 1:5000), or anti-goat IgG-HRP (Santa Cruz, sc-2020, 1:5000). The protein bands were detected by Enhanced ECL AmershamTM prime Western blotting detection reagent (GE Healthcare, RPN2232).

### 2.6. Immunofluorescence Microscopy

Cells were washed twice in PBS, then fixed in freshly prepared 3.7% paraformaldehyde (PFA) in PBS (pH 7.4) for 15 min on ice cube, permeabilized in 0.1% Triton X-100 in blocking solution (3% goat serum plus 0.5% BSA in PBS) for 30 min at room temperature, washed three times (each for 15 min) and left in the blocking solution for 1 h. Cells were then incubated overnight at 4 °C with primary antibodies against Zscan4 (AB4340, Millipore, 1:500), H3K9me3 (ab8898, Abcam, 1:1000), washed three times (each for 15 min), and incubated for 1 h with the secondary antibody Fluor 568 goat anti-rabbit IgG (A11036, MP), diluted 1:200 with blocking solution. Samples were washed, and counterstained with 0.5 µg/mL Hoechst33342 (H1398, MP) in Vectashield mounting medium. Fluorescence was detected and imaged using a Zeiss inverted fluorescence microscope (Axio Imager Z1).

### 2.7. Telomere Quantitative Fluorescence In Situ Hybridization (Q-FISH)

Telomere length and function (telomere integrity and chromosome stability) was estimated by telomere quantitative FISH. Cells were incubated with 0.5 μg/mL nocodazole for 1.5 h to enrich cells at metaphases. Chromosome spreads were made by a routine method. Metaphase-enriched cells were exposed to hypotonic treatment with 75 mM KCl solution, fixed with methanol: glacial acetic acid (3:1) and spread onto clean slides. Telomere FISH and quantification were performed as described previously [25], except for FITC-labeled (CCCTAA) peptide nucleic acid (PNA) probe used in this study. Telomeres were denatured at 80 °C for 3 min and hybridized with telomere specific PNA probe (0.5 μg/mL) (Panagene, Daejeon, Korea). Chromosomes were counter-stained with 0.5 μg/mL DAPI. Fluorescence from chromosomes and telomeres was digitally imaged on a Zeiss microscope with FITC/DAPI filters, using AxioCam and AxioVision software 4.6. For quantitative measurement of telomere length, telomere fluorescence intensity was integrated using the TFL-TELO program (gift kindly provided by P. Lansdorp), and calibrated using standard fluorescence beads.

### 2.8. Telomere Measurement by Quantitative Real-Time PCR (T/S Ratio)

Genome DNA was prepared using E.N.Z. DNeasy Blood & Tissue Kit (QIAGEN, Valencia, CA, USA). Average telomere length was measured from total genomic DNA using a real-time PCR assay [26]. PCR reactions were performed on the iCycleriQ real-time PCR detection system (Bio-Rad, Hercules, CA, USA), using telomeric primers, primers for the reference control gene (mouse 36B4 single copy gene) and PCR settings as previously described [27]. For each PCR reaction, a standard curve was made by serial dilutions of known amounts of DNA. The telomere signal was normalized to the signal from the single copy gene to generate a T/S ratio indicative of relative telomere length. Equal amounts of DNA were used for each reaction. Primers for T/S ration assay are shown in Table S2.

### 2.9. Telomere Chromatid Orientation-Fluorescence In Situ Hybridization (CO-FISH)

CO-FISH assay was performed according to our previous work [24]. Briefly, subconfluent cells were incubated with 10 μM of 5′-bromo-2′-deoxyuridine (BrdU) for 12 h to allow BrdU incorporation for one cell cycle. Nocodazole was added for 2 h prior to cell harvest, and metaphase spreads were prepared by standard cytogenetic method as described above for Q-FISH. Chromosome slides were treated with RNaseA, fixed with 4% formaldehyde, then stained with Hoechst 33258 (0.5 mg/mL) for 15 min and exposed to 365 nm UV light (Stratalinker 1800 UV irradiator) for 40 min. The BrdU-substituted DNA was digested with Exonuclease III (Promega, Madison, WI, USA). The slides were then dehydrated through cold ethanol series and air dried. PNA-FISH was performed with Fluorescein-OO-(CCCTAA)_3_ (Bio-Synthesis, Lewisville, TX, USA). Slides were hybridized, washed, dehydrated, mounted, and counterstained with VectaShield antifade medium (Vector, Peterborough, UK), containing 0.1 mg/mlDAPI. Digital images were captured using a CCD camera on a Zeiss Axio-Imager Z1 microscope equipped with Metasystems Isis software.

### 2.10. Global DNA Methylation Analysis

DNA methylation was analyzed by immunostaining with 5-MeC (NA81, Calbiochem, San Diego, CA, USA) and 5hmC (39769, active motif) antibodies as we previously described [28]. ES cells were dissociated in trypsin–EDTA and washed in PBS twice and fixed in ice-cold 3.7% PFA for 30 min, washed in 0.05% PBT, permeabilized in 0.2% Triton X-100 solution for 30 min. Then, cells were depurinated in 4 N HCl, 0.1% Triton X-100 for 10 min, washed, and then blocked in 2% BSA in PBS. Samples were incubated with mouse anti-5-MeC or anti-5hmC antibody over-night at 4 °C, washed and then incubated with FITC conjugated goat anti-mouse IgG (A-11001, Molecular Probes, 1:500) or FITC conjugated anti-rabbit IgG (554020, BD Biosciences, 1:500) in blocking solution for 1 h at RT, washed, and then ES cells were placed in the fluorescence-activated cell sorting for analysis and sorting by flow cytometry (BD Biosciences, Franklin Lakes, NJ, USA). Cells with immunostaining only with normal mouse or rabbit IgG served as negative controls. Fluorescence distribution and intensity of cell populations following immunostaining with anti-5-MeC and 5hmC antibodies were quantified by FACS analysis. 

### 2.11. Bisulfate Sequencing

WT, G4 Terc^-/-^ ES cells and WT ES cells treated with 1 µM 5-aza-dC for 2.5 days were analyzed for DNA methylation of mouse *Zscan4c* promoter and *Tcstv1*/*Tcstv3* promoter. HeLa and U2 OS cells were used to analyze for DNA methylation of human ZSCAN4 promoter. Briefly, 1 µg of genomic DNA was subject to sodium bisulfite conversion using the EpiTect Bisulfite kit (QIAGEN). Mouse *Zscan4c* promoter and human ZSCAN4 promoter were amplified using the following bisulfite primers. For mouse *Zscan4c* promoter, forward: GATTAGATTTTGAAGAATATATTTTTTGTG; reverse: CCTCCAATATAACAAAACCCTTAAC. For mouse *Tcstv1/3* promoter, forward: TTGGTATATTTGGTGGGTTAAGAAG; reverse: AATCCACAATTCTCCTTCAAAAATA. For human ZSCAN4 promoter, forward: TGGTTTTTATAGGTTTTGTATAGATT; reverse: AAAACTAAAATCCCCTACCAACTTC. The PCR products were cloned into the pEasy-T1 simple vector (Transgene) through TA cloning, and 8–15 colonies were randomly picked and sequenced and analyzed for each sample. 

### 2.12. ChIP-qPCR Analysis

WT and G4 Terc^-/-^ ES cells, HeLa and U2 OS cells were used for ChIP experiment as we previously described [24]. Briefly, cells were fixed with freshly prepared 1% paraformaldehyde for 10 min at room temperature. Cells were then harvested and their nuclei extracted, lysed, and sonicated. DNA fragments were then enriched by immunoprecipitation with 4 µg H3K9me3 (ab8898, Abcam) antibody per ChIP. The eluted protein:DNA complex was reverse-crosslinked at 65 °C overnight. DNA was recovered after proteinase and RNase A treatment. In ChIP-qPCR experiments, the ChIP-enriched DNA was analyzed by real-time PCR using primers for mouse and human Zscan4 promoter regions, β-actin served as negative control, and relative fold changes were normalized to IgG controls. The primers used are in Table S4.

### 2.13. Co-Localization of Telomeresand Human Chromosome 19

Telomeric FISH was performed as previously described [27] with minor modifications. Briefly, after slides were fixed for 2 min in 4% formaldehyde, dehydrated in a series of ethanol (70%, 90%, and 100%) and air dried, they were denatured with 0.5 µg/mL of telomere probe and 2 µL of chromosome 19 probe that specifically hybridizes to 19p13 in 10 µL of hybridization buffer (offered in chr 19 probe kit) (CHR19-10-RE, Empire Genomics) at 73 °C for 3 min and hybridized at 37 °C for 2.5 h. After hybridization, slides were washed twice with 70% formamide, followed by three washes in TBS-Tween (0.08%) buffer. Then slides were dehydrated with a series of ethanol and air dried. Chromosomes were stained with 0.5 µg/mL DAPI. Images were taken at 63× magnification using a Zeiss fluorescence microscope.

### 2.14. Statistical Analysis

Data were analyzed by ANOVA and means compared by Fisher’s protected least-significant difference (PLSD) using the StatView software from SAS Institute Inc. (Cary, NC, USA). Significant differences were defined as *p* < 0.05, 0.01, or lower.

## 3. Results

### 3.1. Late Generation Terc^-/-^ ES Cells Maintains Stably Short Telomere Length during Prolonged Passaging

**Figure 1:**
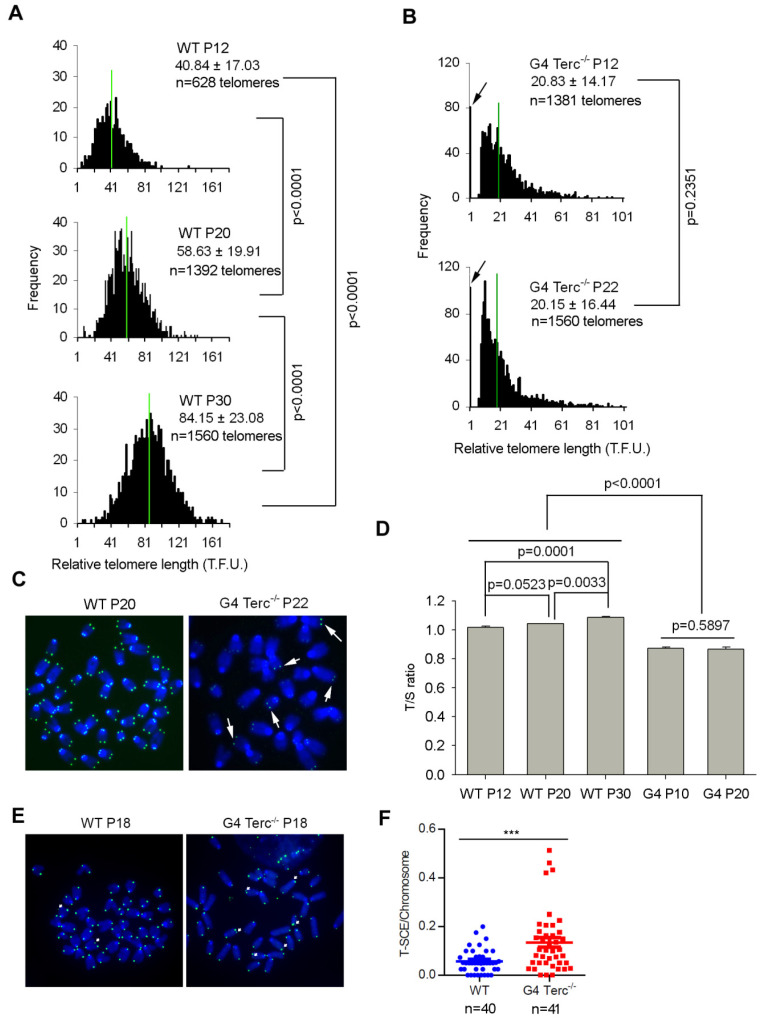
Telomere length dynamics in WT and G4 Terc^-/-^ ES cells at different passages. (**A**,**B**) Distribution histogram of relative telomere length at different passages of WT (**A**) and G4 Terc^-/-^ (**B**) ES cells, as analyzed by telomere Q-FISH and the TFL-TELO software. TFU, arbitrary telomere fluorescence unit. Green lines indicate medium telomere length. The average length ± SD is given in the upper right corner. Arrows indicate frequency of telomere loss. (**C**) Representative telomere-specific PNA-FISH image of WT P20 and G4 Terc^-/-^ P22 ES cells. The G4 Terc^-/-^ ES cells show typical ALT characteristics, with some telomeres longer than others shown with white arrow. (**D**) Relative telomere length of WT and G4 Terc^-/-^ ES cells at various passages shown as T/S ratio. Error bars indicate mean ± SEM (*n* = 3). (**E**) Micrographs showing T-SCE (arrows) by CO-FISH analysis. (**F**) Increased frequency of T-SCE per chromosome in G4 Terc^-/-^ ES cells. *n*, number of metaphase spread images analyzed; ***, *p* < 0.001, compared to controls.

The late fourth generation Terc^-/-^ ES cells (G4) show similar morphological characteristics of WT ES cells, even though they harbor a slower proliferation rate [7]. Short telomeres were maintained in G4 Terc^-/-^ ES cells during prolonged passaging (Figure 1). Telomere quantitative fluorescence in situ hybridization (QFISH) analysis of telomere length dynamics in WT ES cells shows that telomeres elongated dramatically from passage 12 to 20 and to 30 (*p* < 0.0001) (Figure 1A), consistent with previous reports that telomeres lengthened during passaging [29,30,31]. In contrast, G4 Terc^-/-^ ES cells maintained their telomeres without notable shortening between passages 12 and 22 (*p* = 0.2351, 20.83 ± 14.17 TFU vs. 20.15 ± 16.44 TFU) (Figure 1B), but displayed increased frequency of signal-free ends, indicating telomere loss (Figure 1B). Notably, G4 Terc^-/-^ ES cells showed typical ALT characteristics of heterogeneous telomeres, with some telomeres longer than others (indicated by white arrows), unlike telomeres of WT ES cells (Figure 1C). The telomere QFISH data on telomere elongation in WT and telomere maintenance of G4 Terc^-/-^ ES cells was further validated by quantitative real-time PCR shown as T/S ratio [26] (Figure 1D), suggesting a telomerase-independent mechanism of telomere maintenance might be triggered by short telomeres in G4 Terc^-/-^ ES cells. Indeed, the frequency of telomere sister-chromatid exchange (T-SCE) events, as analyzed by chromosome orientation FISH (CO-FISH), increased remarkably in G4 Terc^-/-^ ES cells (Figure 1E,F). These data suggest that telomerase-independent mechanisms are responsible for rescuing telomeres that would otherwise shorten in G4 Terc^-/-^ ES cells without telomerase.

### 3.2. Up-Regulation of Zscan4 Is Implicated in Telomere Maintenance of G4 Terc^-/-^ ES Cells

**Figure 2:**
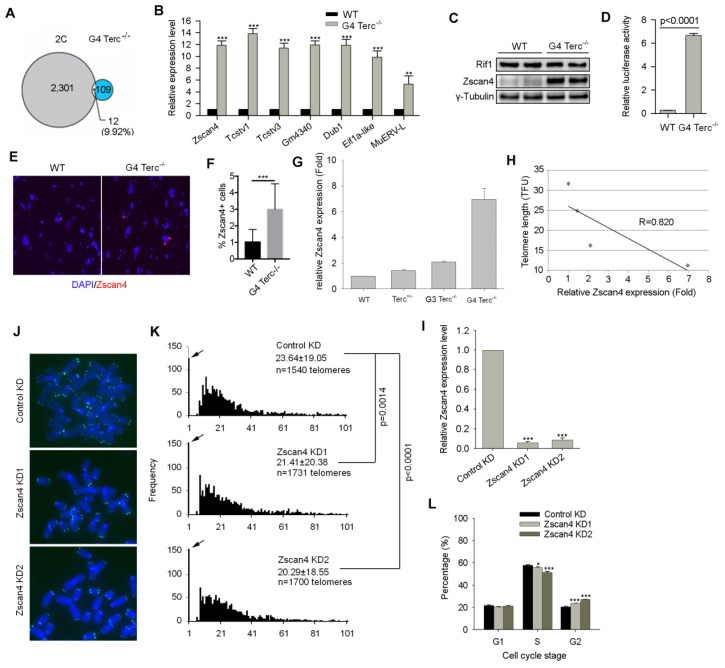
High expression level of Zscan4 contributes to telomere maintenance in G4 Terc^-/-^ ES cells. (**A**) A Venn diagram shows the overlap between 2C-genes and genes upregulated in G4 Terc^-/-^ ES cells (2C-gene data [32] and G4 Terc^-/-^ data [7] were analyzed). (**B**) Two cell-specific genes *Zscan4*, *Tcstv1*, *Tcstv3*, *Gm4340*, *Dub1,* *Eif1a-like,* and MuERV-L are all up-regulated in G4 Terc^-/-^ ES cells. *Zscan4*, *Tcstv1,* and *Tcstv3* are located at sub-telomeric regions and *Gm4340* is located at peri-centromeric region in mouse genome. (**C**) Western blotting analysis of Zscan4 and Rif1 protein levels between WT and G4 Terc^-/-^ ES cells. Two technical repeats were included. (**D**) Relative luciferase activity of Zscan4c promoter between WT and G4 Terc^-/-^ ES cells. (**E**) Representative Zscan4 immunofluorescence images of Zscan4^+^ cells in WT and G4 Terc^-/-^ ES cells. (**F**) Relative percentage of Zscan4^+^ cells. Error bars indicate mean ± SEM. (**G**) Comparison of Zscan4 relative expression levels by qPCR analysis of WT, Terc^+/-^, G3, and G4 Terc^-/-^ ES cells. (**H**) Relative telomere length shown as TFU of ESCs is highly (R = 0.820) correlated with Zscan4 expression levels among different cells lines. The telomere length data used for correlation analysis were showed in our previous paper [7]. (**I**) Zscan4 mRNA levels were reduced by shRNA sequences targeting Zscan4 gene. (**J**) Representative telomere specific PNA-FISH images of control and Zscan4 KD G4 Terc^-/-^ ES cells. (**K**) Histogram shows telomere distribution and relative lengths following Zscan4 KD by telomere QFISH. Frequency of telomere end free-signals is shown on Y-axis indicated by arrows. Upper right-hand corner, the average length ± SD. (**L**) Cell cycle progression analysis shows reduced S phase and increased G2 phase of G4 Terc^-/-^ ES cells following knockdown of *Zscan4*. Error bars indicate mean ± SEM (*n* = 3). *, *p* < 0.05; **, *p* < 0.01; ***, *p* < 0.001, compared to controls.

Interestingly, in comparison with global gene expression profile of WT ES cells [7], G4 Terc^-/-^ ES cells showed upregulation of two-cell (2C) genes including *Tcstv1*, *Tcstv3*, *Gm4340*, *Dub1*, *Eif1a-like* (*Gm2022*), and *MuERV-L* [32], and particularly, *Zscan4*, which is involved in telomere recombination and elongation [16] (Figure 2A), validated by qPCR here (Figure 2B). Western blotting analysis also verified the up-regulation of Zscan4 protein level in G4 Terc^-/-^ ES cells (Figure 2C). Notably, Rif1 protein level was not changed noticeably (Figure 2C), which was involved in negative regulation of Zscan4 expression by our previous report [24]. Consistently, *Zscan4* promoter activity analyzed by dual luciferase assay elevated dramatically in G4 Terc^-/-^ ES cells compared with WTES cells (*p* < 0.0001) (Figure 2D), another evidence supporting the elevation of *Zscan4* expression in G4 Terc^-/-^ ES cells with short telomeres. Moreover, the Zscan4^+^ ES cell population in G4 Terc^-/-^ ES cell cultures increased remarkably (Figure 2E,F). However, Terc^+/-^ ES cells with sufficient telomere lengths and pluripotency [7], expressed *Zscan4* at levels similar to those of WT ES cells, in contrast to much higher *Zscan4* levels of G4 Terc^-/-^ ES cells with short telomere (Figure 2G), indicating highly correlation between Zscan4 expression levels and telomere lengths (Figure 2H). These results together further suggest that short telomeres lead to excessive activation of *Zscan4*. Consistently, a previous report with live cell imaging technique showed that *Zscan4* is activated after telomere shortening in mESCs to recover the shortened telomeres during extended cell cycles [33].

To test whether *Zscan4* is implicated in telomere maintenance of G4 Terc^-/-^ ES cells, we depleted *Zscan4* by specific shRNAs targeting *Zscan4* gene (targeting all *Zscan4* isoforms) (Table S1). *Zscan4* mRNA levels were reduced to minimal after *Zscan4* knockdown (KD) (Figure 2I). Telomeres of *Zscan4* KD G4 Terc^-/-^ ES cells were shorter than those of control KD G4 Terc^-/-^ ES cells analyzed by telomere QFISH (Figure 2J,K). Furthermore, cell cycle progression was reduced as evidenced by shorter S phase but longer G2 phase following *Zscan4* depletion compared to control KD G4 Terc^-/-^ mESCs (Figure 2L). Collectively, *Zscan4* was excessively activated in G4 Terc^-/-^ ES cells with short telomeres in the absence of telomerase activity and likely responsible for ALT-mediated telomere elongation to compensate for telomere shortening.

### 3.3. DNA Methylation and H3K9me3 Regulate Zscan4 of G4 Terc^-/-^ ES Cells

We searched for the mechanisms underlying up-regulation of *Zscan4* in G4 Terc^-/-^ ES cells with very short telomeres. Further analysis of the microarray data in Figure 2A identified that many of the up-regulated genes in G4 Terc^-/-^ ES cells, including *Zscan4*, *Tcstv1*, *Tcstv3,* and *Gm4340*, are located at subtelomeric or pericentromeric regions. *Zscan4* gene clusters are located at subtelomeric regions of short p arm of chromosome 7 (Figure S1A). Telomeres and subtelomeres are densely enriched with repressive DNA and histone modifications (e.g., H3K9me3) that regulate mammalian telomere length negatively [9,34]. We hypothesized that the repressive DNA methylation and histone modifications may inhibit gene expression at subtelomeres including *Zscan4*, and *Tcstv1/3*, and that reduction of the repressive DNA and histone modifications de-represses *Zscan4* and contributes to telomere maintenance by activating recombination-based telomere elongation mechanisms in ES cells with short telomeres.

**Figure 3:**
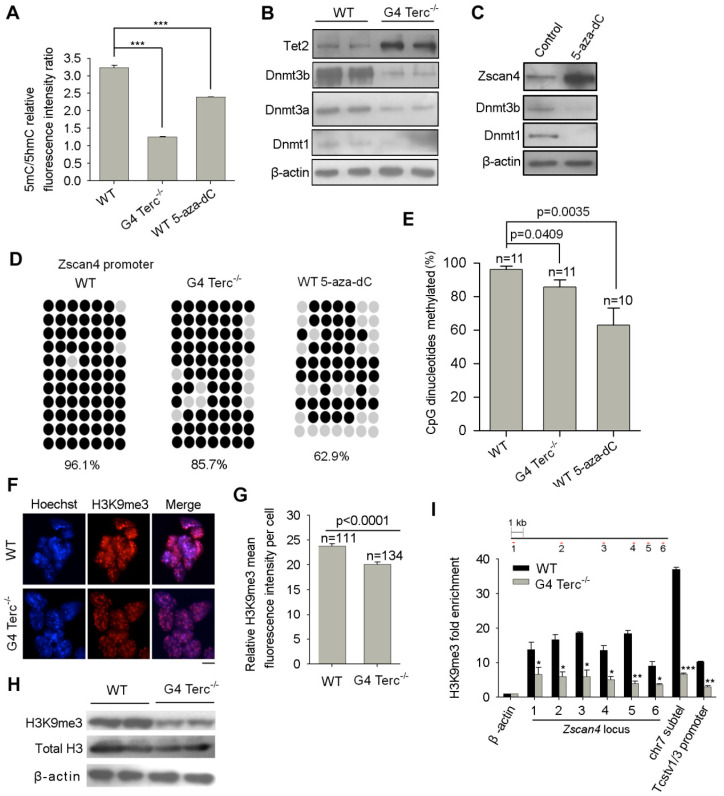
Reduction of DNA methylation and H3K9me3 occupancy at Zscan4 promoter regions in G4 Terc^-/-^ ES cells with short telomeres. (**A**) Relative quantity (RQ) of DNA methylation levels among WT, G4 Terc^-/-^, and 5-aza-dC treated WT ES cells (1 µM for 2.5 days). Quantification was made by quantifying mean fluorescence intensity of cell populations following immunostaining with anti-5mC and 5hmC antibodies and analyzed by FlowJo software. Error bars indicate mean ± SEM (*n* = 3). ***, *p* < 0.001, compared to WT controls. (**B**) Western blotting shows decreased Dnmt1, Dnmt3a, Dnmt3b, and increased Tet2 protein levels in G4 Terc^-/-^ ES cells, respectively. Two technical repeats were included. (**C**) Western blotting shows decreased Dnmt1, Dnmt3b protein levels after 5-aza-dC treatment (1 µM for 2.5 days) in WT ES cells. (**D**) Bisulfate sequencing analysis of CpG dinucleotides of mouse Zscan4c proximal promoter regions among WT, G4 Terc^-/-^ and 5-aza-dC treated WT ES cells (1 µM for 2.5 days). Open circles, unmethylated; closed circles, methylated. (**E**) Quantification of bisulfate genomic sequencing results of 10–11 independent clones (n) at Zscan4c proximal promoter. Note the decrease in methylated CpGs at Zscan4c proximal promoter in G4 Terc^-/-^ and 5-aza-dC treated WT ES cells compared with WT ES cells. Error bars indicate mean ± SEM. (**F**) Representative images shows immunofluorescence staining of heterochromatic H3K9me3 (red). Nuclei stained with Hoechst33342 (blue) show brighter fluorescence signal in the heterochromatin. Scale bar, 10 μm. (**G**) Quantification of relative H3K9me3 mean fluorescence intensity between WT and G4 Terc^-/-^ ES cells using ImageJ software. Randomly chosen cells from 9–10 fields of view were counted. n, number of cells counted. (**H**) Western blotting analysis shows the decreased inhibitive histone modification H3K9me3 in G4 Terc^-/-^ ES cells. (**I**) ChIP-qPCR analysis of H3K9me3 occupancy at six proximal locations upstream to *Zscan4c* promoter, sub-telomere further upstream to *Zscan4c* promoter of chr7 and *Tcstv1*/*Tcstv3* promoter at subtelomere of chr13. Relative fold enrichment was normalized to rabbit IgG ChIP signals at the same regions, and the β-actin locus served as negative controls. Error bars indicate mean ± SEM. *, *p* < 0.05; **, *p* < 0.01; ***, *p* < 0.001, compared to controls.

We first assessed the global DNA methylation levels between WT and G4 Terc^-/-^ ES cells. FACS analysis showed reduced global 5mC/5hmC relative fluorescence intensity in G4 Terc^-/-^ ES cells compared with WT ES cells (Figure 3A and Figure S1B). DNA methylation levels could be balanced by DNA methyltransferases (DNMT enzymes) and 5-methylcytosine oxidases (TET enzymes). All three DNMT enzymes were decreased while Tet2 was increased in G4 Terc^-/-^ ES cells as analyzed by immunoblot assays (Figure 3B). Quantitative PCR analysis also confirmed the downregulation of *Dnmt3a* and *Dnmt3b*, up-regulation of *Tet2*, in G4 Terc^-/-^ ES cells, respectively (Figure S1C). Interestingly, *Dnmt1* mRNA level was not changed despite significant reduction at protein level in G4 Terc^-/-^ ES cells (Figure 3B and Figure S1C), suggesting post-transcriptional regulation of Dnmt1 involved in G4 Terc^-/-^ ES cells. Consistently, *Tert* KO ES cells with short telomeres also showed the reduction of both Dnmt3a and Dnmt3b protein levels and global DNA methylation [35]. Previously, we have demonstrated that over-expression of Tbx3 in mouse ES cells led to decreased Dnmt3b and increased Tet2 protein levels, respectively, synergistically resulting in reduced DNA methylation level and elevated Zscan4 expression level [28]. Indeed, *Tbx3* was upregulated in G4 Terc^-/-^ ES cells compared to WT ES cells (Figure S1C).

In support of the inhibitory effects of DNA methylation on *Zscan4*, ES cells were treated with DNA methyltransferase inhibitor, 5-aza-deoxycytidine (5-aza-dC), and showed decreased Dnmt1 and Dnmt3b protein levels (Figure 3C). Consistently, the global DNA methylation was decreased significantly following 5-aza-dC treatment (Figure 3A and Figure S1B). Furthermore, bisulfate sequencing analysis showed that *Zscan4c* promoter was heavily methylated in WT ES cells and decreased slightly in G4 Terc^-/-^ ES cells, and decreased further in 5-aza-dC treated ES cells (Figure 3D,E). Moreover, significantly decreased DNA methylation also was found at *Tcstv1/3* promoter (*Tcstv1* and *Tcstv3* genes share the same promoter sequence) in G4 Terc^-/-^ ES cells (Figure S1D,E).

Next, we compared the histone modifications between WT and G4 Terc^-/-^ ES cells. Heterochromatin marked by H3K9me3 foci was reduced in G4 Terc^-/-^ ES cells, compared with WT ES cells as analyzed by both immunofluorescence and immuoblot assays (Figure 3F–H). We have previously demonstrated that *Zscan4* was repressed by H3K9me3 binding at its promoter [24]. Thus, we asked whether the reduced H3K9me3 occupancy at specific genomic loci was within or near the proximity of *Zscan4c* loci in G4 Terc^-/-^ ES cells, which could account for the de-repression of *Zscan4*. We performed ChIP-qPCR analysis to determine the H3K9me3 occupancy at *Zscan4c* loci and found that H3K9me3 level was decreased in six proximal locations upstream to *Zscan4c* promoter and subtelomeres of chromosome 7 further upstream to *Zscan4c* locus (Figure 3I). Moreover, H3K9me3 level was also decreased in *Tcstv1*/*Tcstv3* proximal promoter at subtelomeres of chromosome 13 (Figure 3I), also in line with the upregulation of *Tcstv1/Tcstv3* expression in G4 Terc^-/-^ ES cells (Figure 2A,B). Taken together, short telomeres reduce DNA methylation and H3K9me3 levels and occupancy at subtelomeres and telomeres of G4 Terc^-/-^ ES cells and thus de-repress *Zscan4* expression at subtelomeres. 

### 3.4. Inhibition of Repressive DNA and H3K9 Methylation Up-Regulates Zscan4 and Elongates Telomeres 

**Figure 4:**
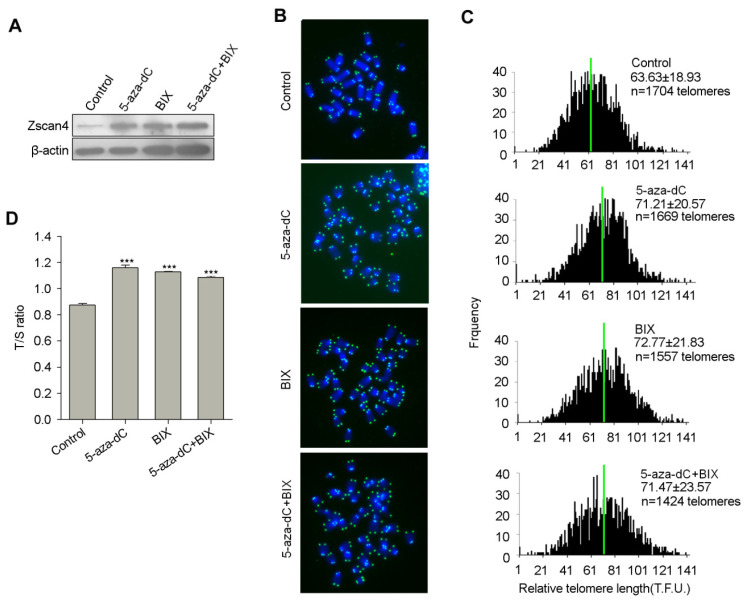
Inhibition of DNA methylation and H3K9 methylation elevates Zscan4 expression levels and elongates telomere lengths. (**A**) Western blotting analysis shows increased Zscan4 protein levels after treatment with small molecules. 5-aza-dC, DNA methyltransferases (DNMTs) inhibitor; BIX-01294, histone lysine methyltransferases (HMTases) inhibitor. WT ES cells were treated with 1 µM 5-aza-dC, or 1 µM BIX-01294, or both for 2.5 days. (**B**) Telomere specific PNA-FISH image of WT ES cells after treatment with 5-aza-dC, or/and BIX-01294 for four passages. (**C**) Distribution histogram of relative telomere length of WT ES cells after different treatments, analyzed by telomere Q-FISH and the TFL-TELO software. Green lines indicate medium telomere length. The average length ± SD is given in the upper right corner. Arrows indicate frequency of telomere loss. (**D**) Relative telomere length of WT ES cells after different treatments shown as T/S ratio. Experiments performed in (**B**–**D**) were treated with 1 µM 5-aza-dC, or 1 µM BIX-01294, or both, for four passages, 10 days. After the treatments, genomic DNA and chromosome spreads were used for T/S ratio and QFISH assays for telomere length measurement, respectively. Error bars indicate mean ± SEM (*n* = 3). ***, *p* < 0.001, compared to controls.

Decreased telomeric repressive DNA and histone modifications (e.g., H3K9me3) relieve the inhibition of *Zscan4* at subtelomeric heterochromatin regions, contributing to telomere maintenance by activating telomere recombination. Next, we tested whether inhibiting repressive DNA and H3K9 methylation by small molecules can also relieve *Zscan4* inhibition and elongate telomere length. Treatment of ES cells with DNA methyltransferase inhibitor 5-aza-dC, or histone H3K9methyltransferase inhibitor BIX-01294, or both, all significantly elevated Zscan4 protein levels (Figure 4A). Consequently, treatments with 5-aza-dC, orBIX-01294, or both, all significantly elongated telomere length as analyzed by QFISH analysis (Figure 4B,C). The telomere QFISH on telomere elongation data was further validated by quantitative real-time PCR shown as T/S ratio (Figure 4D). These data further support that epigenetic modification regulates expression of heterochromatic genes, and *Zscan4* at subtelomeres in this case. 

### 3.5. ZSCAN4 Depletion in Telomerase-Deficient Human ALT U2 OS Cancer Cells Shortens Telomeres

ALT positive U2 OS cancer cells are characterized by promyelocytic leukemia (PML) bodies and high frequency of T-SCE, indicative of telomere recombination [36,37,38], and show extremely long telomeres: even longer than those of human ES cells. However, these ALT cells have minimal or no telomerase activity, in contrast to hES cells [39,40]. Given the similarity of telomerase-deficient mESCs and ALT human cancer cells in maintaining telomeres independent of telomerase involvement for cellular proliferation, we hypothesized that ZSCAN4 may also be involved in telomere maintenance of human ALT U2 OS cancer cells. 

**Figure 5:**
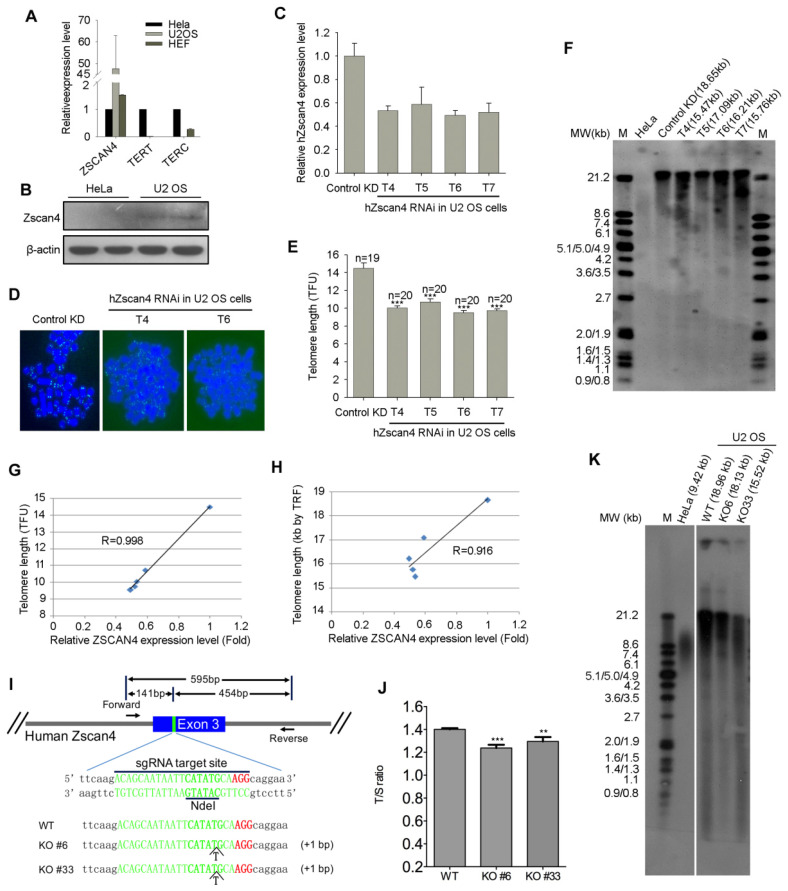
Depletion of ZSCAN4 in ALT U2 OS cells shortens telomere length. (**A**,**B**) Human ZSCAN4 is highly up-regulated in ALT positive U2 OS cells compared to ALT negative HeLa cells, analyzed by qPCR (**A**) and Western blotting (**B**). (**C**) Stable knockdown efficiency of ZSCAN4 analyzed by qPCR with four different ZSCAN4RNAi sequences. (**D**) Representative telomere specific PNA-FISH image of control and stable ZSCAN4 KD U2 OS cells. Image of control KD cells shows telomere length heterogeneity, typical ALT cell characteristics of human cancer cells. (**E**) Quantitative telomere length of control and ZSCAN4 stable KD U2 OS cells. The error bars indicate mean of telomere length ± SEM (*n* = 19–20 chromosome spreads). TFU, telomere fluorescence unit. ***, *p* < 0.0001, compared to control KD. (**F**) TRF analysis of control and stable ZSCAN4 KD U2 OS cells. A volume of 1.5 μg of DNA was digested and separated on a 0.8% agarose gel. M indicates a set of DNA size markers, and MW refers to DNA sizes in kilobases. Telomere length was measured using Telomeric version 1.2 software, and the median telomere length of each lane was given upper the gel. (**G**,**H**) Human ZSCAN4 expression levels after RNAi with different targeting sequences are highly correlated with relative telomere length analyzed by QFISH (R = 0.998) (**G**) and TRF assay (R = 0.916) (**H**). (**I**) Targeted disruption of ZSCAN4 gene by CRISPR/Cas9 technology in U2 OS cells. The designed sgRNA targeting human ZSCAN4 gene exon 3 is underlined, and the protospacer-adjacent motif (PAM) sequence labeled in red. By sequencing, two independent homologous KO clones were identified by insertion of a T at the restriction site. (**J**,**K**) ZSCAN4 KO in U2 OS cells shortens telomere length as analyzed by real time qPCR shown as T/S ratio (**J**) and TRF assay (**K**). Telomere length shown in (**J**) and (**K**) represents mean telomere length. Error bars indicate mean ± SEM (*n* = 3). **, *p* < 0.01, ***, *p* < 0.001, compared to controls.

First, we compared the gene expression levels of ZSCAN4 in the well-defined telomerase-positive/ALT negative HeLa cells, telomerase negative/ALT positive U2 OS cells and telomerase/ALT double deficient fibroblast cells (negative control), and found that ZSCAN4 was highly up-regulated in U2 OS cells as analyzed by qPCR (Figure 5A). Western blotting analysis also showed dramatically increased human ZSCAN4 protein level in U2 OS cells (Figure 5B). 

To test whether ZSCAN4 is involved in telomere maintenance of U2 OS cells, we stably knocked-down ZSCAN4 with lentivirus-mediated RNA interference with high efficiency (Figure S2) using four different shRNA sequences targeting ZSCAN4 gene (Table S1). ZSCAN4 expression levels were decreased by the four shRNA sequences as analyzed by qPCR (Figure 5C). Telomeres shortened by telomere Q-FISH analysis of ZSCAN4 knockdown in U2 OS cells, using four different shRNA sequences, compared with knockdown controls (Figure 5D,E). TRF assay also confirmed the telomere shortening following ZSCAN4 KD (Figure 5F). Notably, the telomere shortening rate as analyzed by both Q-FISH and TRF assays is positively correlated with ZSCAN4 KD efficiency in ALT-U2 OS cells (Figure 5G,H). We further confirmed the telomere shortening by CRISPR-Cas9 mediated deletion of ZSCAN4 in U2 OS cells (Figure 5I–K). These data suggest that ZSCAN4-mediated telomerase-independent mechanisms can also be responsible for telomere length maintenance of human ALT positive U2 OS cancer cells.

### 3.6. Human ZSCAN4 Is Also Repressed by Epigenetic Marks at Its Promoter

**Figure 6:**
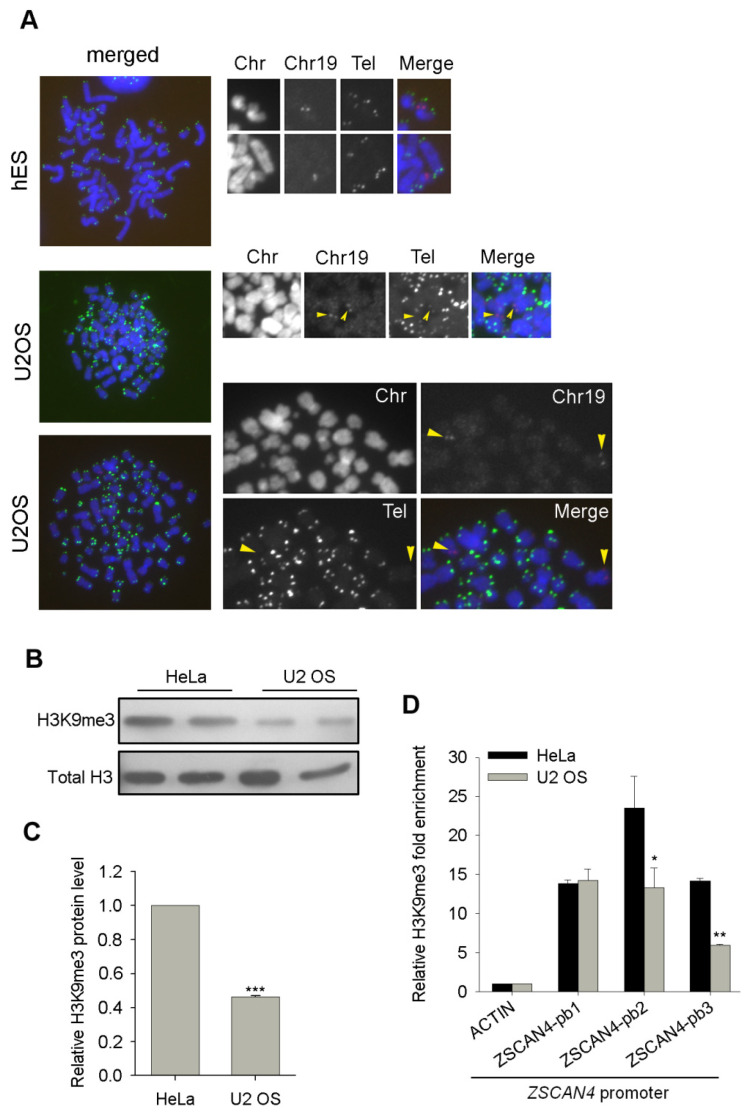
Heterochromatin de-repression activates ZSCAN4 in ALT-U2 OS cells. (**A**) Co-localization of telomeres and ZSCAN4 gene region on chromosome 19 in telomerase-positive hESCs and telomerase negative U2 OS cells. Orange arrowheads indicate telomere signals of chromosome 19 where ZSCAN4 gene locates. (**B**) Western blotting analysis of H3K9me3 levels between HeLa and U2 OS cells. (**C**) Relative protein quantity of H3K9me3 normalized to β-actin by Bio-Rad Quantity One software. Error bars indicate mean ± SEM (*n* = 2). ***, *p* < 0.001, compared to controls. (**D**) ChIP-qPCR analysis of H3K9me3 occupancy at 3 proximal locations upstream to ZSCAN4 promoter at subtelomeric regions of chromosome 19. Relative fold enrichment was normalized to rabbit IgG-ChIP signals at the same regions, and the β-actin locus served as negative controls. ‘pb’ stands for promoter binding site. Error bars indicate mean ± SEM (*n* = 2). *, *p* < 0.05, **, *p* < 0.01, compared to controls.

Of note, the ZSCAN4 gene also locates at subtelomeric region of the p arm of chromosome 19 (Figure S3A). Interestingly, the telomere signal intensity in the p arm of chromosome 19 where the ZSCAN4 gene located was much weaker compared with the q arm of chromosome 19 in telomerase-deficient U2 OS cells (Figure 6A), which was not seen in telomerase-positive hES cells with similar telomere signal intensity between the p and q arm of chromosome 19 (Figure 6A). It is to note that short telomeres following telomerase-deficiency were coupled with reduced repressive DNA and H3K9me3 methylation marks in Terc^-/-^ MEF cells [13] and in G4 Terc^-/-^ ES cells in the present study (Figure 3), suggesting that ZSCAN4 may also be epigenetic regulated by repressive DNA methylation and H3K9me3 in U2 OS cells. 

**Figure 7:**
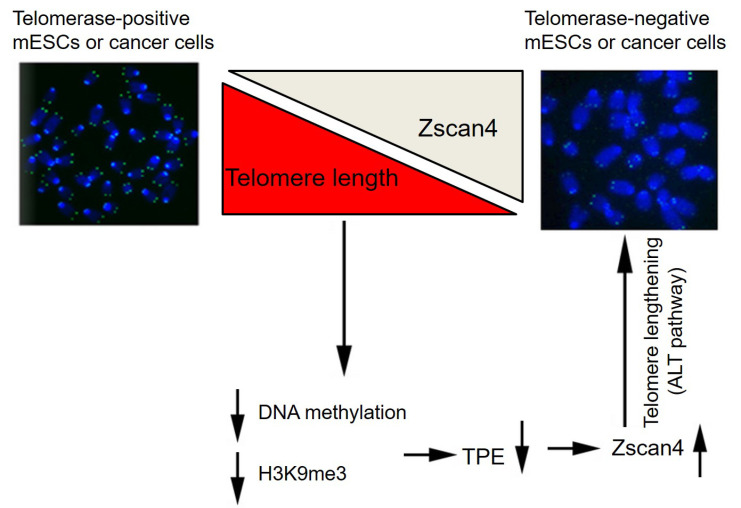
Proposed model of telomere length homeostasis shared in telomerase-deficient late generation mESCs and ALT-U2 OS cells. The telomerase-deficient mouse ES cells and ALT-U2 OS cancer cells share similar mechanisms to maintain telomere homeostasis. *Zscan4* is activated due to reduced TPE associated with shortened telomeres at subtelomeric *Zscan4* loci in those cells. The up-regulation of *Zscan4* activates ALT mechanisms, ensuring telomere homeostasis and the capacity of those cells to proliferate for many passages without telomerase activity.

Bisulfate sequencing analysis showed only minimal difference in DNA methylation levels at ZSCAN4 promoter between HeLa and U2 OS cells (Figure S3B). However, H3K9me3 level was decreased dramatically in U2 OS cells by immunoblot analysis (Figure 6B,C). Furthermore, ChIP-qPCR analysis also showed significant reduced H3K9me3 occupancy at human ZSCAN4 promoter regions (Figure 6D). The above data suggests that human ALT U2 OS cancer cells also may maintain their telomeres in the absence of telomerase activity by reduced epigenetic silencing at subtelomeres and telomeres. Taken together, our results demonstrate that the reduced repressive epigenetic marks-mediated depressed heterochromatin silencing activates Zscan4 expression, which triggers telomere elongation and contributes to telomere maintenance in telomerase-deficient late generation mESCs and ALT U2 OS cancer cells. The positive feedback loop of reduced epigenetic marks/Zscan4 activation/telomere elongation axis ensures telomere homeostasis and the capacity of those cells to proliferate for many passages without telomerase activity (Figure 7).

## 4. Discussion

Telomere length maintenance is essential for indefinite proliferation of mouse ES cells and cancer cells. Very short telomeres could impair functional cellular activities, leading to cell proliferation crisis, cell senescence, and aging [6]. The late generation telomerase-deficient mouse ES cells and telomerase-deficient human ALT cancer cells are able to propagate for numerous passages [7,41,42]. However, how telomeres are maintained in those cells and the mechanisms responding for the telomere maintenance are unclear. Based on our data, we propose that *Zscan4*, which is highly upregulated in those telomerase-deficient late generation mouse ES cells and human ALT U2 OS cells, contributes to telomere maintenance of those cells without telomerase activities. 

*Zscan4* was originally found to specifically express in two-cell embryos and transiently in sporadic ES cells (1–5%) at any given time [16,17], and to lengthen telomeres by recombination-based mechanisms in mouse ES cells [16]. In the case of cancer cells, ZSCAN4 was recently demonstrated to highly express in human head and neck squamous cell carcinoma and to play important roles in the maintenance of cancer stem cell phenotype [43], possibly through the enhancement of telomere maintenance. In the current study, we show that telomere lengths of late generation G4 Terc^-/-^ ES cells maintain stable levels while telomeres of WT ES cells lengthen remarkably during prolonged passaging. Shorter telomeres of G4 Terc^-/-^ ES cells coincide with higher expression of *Zscan4*. In contrast, Terc^+/-^ ES cells with telomerase, despite haplo-insufficiency, show much longer telomeres and expression levels of *Zscan4* similar to WT ES cells, supporting the notion that shorter telomeres could reduce TPE and thus activate *Zscan4* expression. Coincidently, *Zscan4* is localized at the subtelomeres of both mouse and human chromosomes, suggesting that activation of *Zscan4* in both species is regulated by TPE. Interestingly, we previously demonstrated that Zscan4 facilitates telomere elongation by promoting global DNA demethylation in 2C-like mouse ES cells [15]. Moreover, we also showed in the current study that *Zscan4* upregulation and telomere elongation were pronounced after treatment with DNA methyltransferase inhibitor 5-aza-dC (Figure 4). Thus, Zscan4 forms a positive feedback loop to activate itself and promote telomere elongation by inducing DNA hypomethylation. Remarkably, telomeres lengthen rapidly in the early cleavage embryos, progressively in company with global DNA demethylation [27], while *Zscan4* is only expressed in two-cell embryos. Surprisingly, there is no detectable telomerase activity in fertilized zygotes and preimplantation embryos and, telomerase is only reactivated after blastocyst stage [27], suggesting other mechanisms responsible for the telomere elongation beyond the two-cell stage.

Furthermore, specific inhibition of H3K9 methylation results in significant increases in *Zscan4* and telomere elongation, suggesting that loss of repressive histone H3K9me3 may be responsible for ALT cells. By genome-wide epigenetic screen, at the megabase scale, a single feature—levels of H3K9me3—represents major histone modification that can account for more than 40% of mutation-rate variation in human cancer cells [44]. Compared with telomerase-positive HeLa cells, H3K9me3 modification also decreased at human ZSCAN4 promoter regions, located at subtelomeres of the p arm of human chromosome 19 with weak telomere signal intensity, in telomerase-deficient-ALT positive U2 OS cells. Coincidently, ZSCAN4 was highly expressed in telomerase-deficient-ALT positive U2 OS cells compared with telomerase-positive HeLa cells. Moreover, knockdown and Knockout of human ZSCAN4 in U2 OS cells significantly shortened telomere length, suggesting human ZSCAN4 also functions in the contribution of telomere maintenance in human ALT cancer cells without telomerase activity. 

However, the exact mechanisms on how Zscan4 facilitates telomere elongation in both mouse ESCs and human ALT-U2 OS cancer cells remain to be elucidated. Interestingly, it has been shown that TERRAs, the telomeric noncoding RNAs transcribed from subtelomeric promoters by RNA polymerase II, destabilize telomere integrity to initiate break-induced replication (BIR) in human ALT cancer cells [45]. Remarkably, there is ample evidence that BIR processes re-elongate damaged telomeres occurring in the G2 and M phases of the cell cycle through DNA polymerase delta accessory subunits POLD3 and POLD4 [46,47,48,49]. Thus, TERRA transcription elevation results in alternative telomere lengthening through BIR processes. Notably, TERRA expression is inhibited by the repressive epigenetic marks at subtelomeres and, reduction of repressive H3K9me3 and DNA methylation modifications alleviate the repression of TERRA expression [50,51]. Moreover, telomere length negatively regulates TERRA expression. In somatic and ES cells, short telomeres, which are associated with decreased repressive epigenetic marks [9], promote TERRA expression [50], while telomere elongation decreases TERRA molecule levels [51]. Therefore, it is possible that Zscan4 might promote telomere recombination and elongation by upregulating TERRA expression by modulating subtelomeric DNA demethylation, leading to the activation of BIR processes to elongate telomeres. It will be of great interest to investigate this possibility in the future.

Anti-telomerase therapy provokes ALT and other adaptive mechanisms in telomerase-positive cancers [52,53], which account for 85–90% of human cancers, limiting its potential therapeutic usage in clinical medicine. Telomerase-depletion of telomere short cells indeed can induce cell senescence and cell death, but the prolonged depletion of telomerase leads to resistance and activation of ALT [52]. Thus, ALT has the potential to function as an escape mechanism for cells undergoing anti-telomerase therapies, elucidation of the molecular details of the ALT pathway is critical if we are to target these tumors [54]. Combination of anti-telomerase and anti-ALT therapies may shed promising light to anti-cancer battle. Although the regulators underpinning ALT induction and maintenance are poorly understood, recent evidence suggests that SLX4IP and RAD51AP1 are two essential regulators of ALT telomere maintenance in ALT cancer cells [55,56]. Further elucidation of mechanisms involved in ALT telomere lengthening remains to be determined. Our studies proved that human ZSCAN4 gene was highly upregulated in ALT-U2 OS cells, and depletion of ZSCAN4 by shRNA sequences and CRISPR-Cas9 mediated deletion shorten telomeres in ALT-U2 OS cells.

Together, the telomerase-deficient late generation mESCs and ALT-U2 OS cancer cells harbor a similar mechanism to contribute to telomere maintenance for unlimited self-renewal in the absence of telomerase activity. Our data indicate that ZSCAN4 might be a useful target to complement cancer therapy using telomerase inhibitors. 

## Data Availability

The original contributions presented in the study are contained within this article and in the Supplementary Materials, and further inquiries can be directed to the corresponding authors.

## Supplementary Materials

The following supporting information can be downloaded at: https://www.mdpi.com/article/10.3390/cells11030456/s1, Figure S1: DNA methylation level and occupancy at *Tcstv1/3* promoters in G4 Terc^-/-^ mESCs, Figure S2: ALT U2 OS cells were infected with human ZSCAN4 RNAi lentivirus with high infection efficiency, Figure S3: No noticeable difference in DNA methylation occupancy at human ZSCAN4 promoter between HeLa and U2 OS cells, Table S1: 19 nucleotide sequences for RNA knockdown, Table S2: Primers for qPCR analysis of telomere length, Table S3: Primers used for real-time PCR analysis, Table S4: ChIP-qPCR primers of proximal regions of mouse *Zscan4c* promoter and human ZSCAN4 promoter.
