# Supplementary material for: Zscan4 Contributes to Telomere Maintenance in Telomerase-Deficient Late Generation Mouse ESCs and Human ALT Cancer Cells
Source: Cells. 2022 Jan 28;11(3):456. doi: 10.3390/cells11030456 (PMC8834411; doi:10.3390/cells11030456)
Supplement: Supplementary file 1 [file cells-11-00456-s001.zip › cells-1563517-supplementary.pdf]

Supplementary Materials

Zscan4 Contributes to Telomere Maintenance in Telomerase-Deficient Late Generation Mouse ESCs and Human ALT Cancer Cells

Jiameng Dan, Zhongcheng Zhou, Fang Wang, Hua Wang, Renpeng Guo, David L. Keefe and Lin Liu

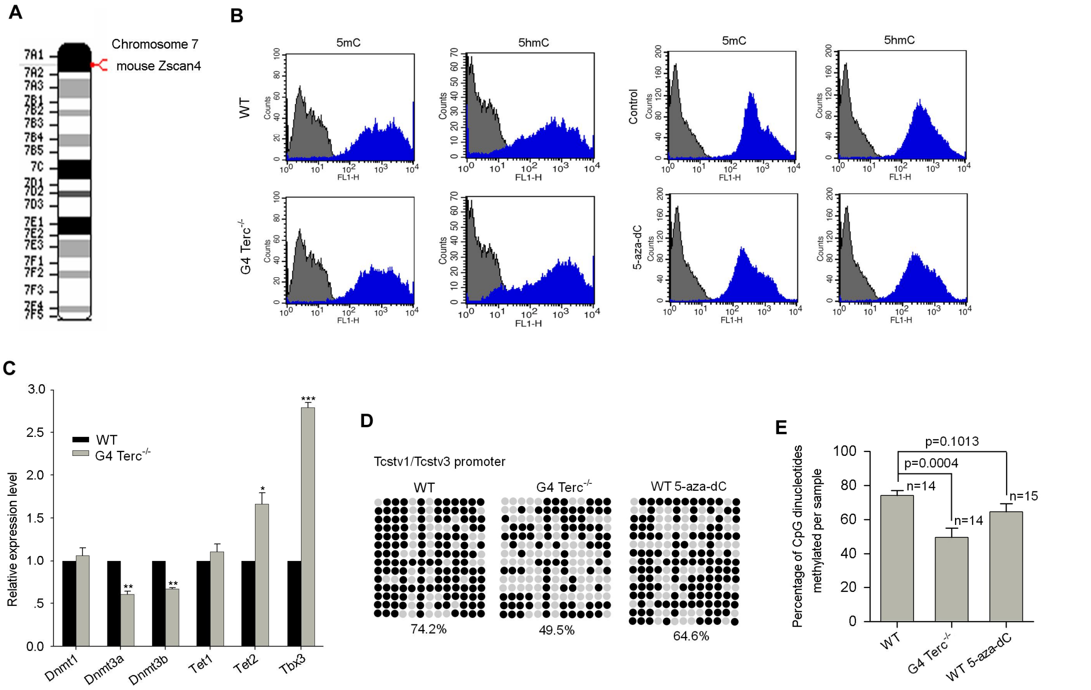

Figure S1. DNA methylation level and occupancy at *Tcstv1/3* promoters in G4 Terc$^{-/-}$ mESCs. **(A)** Subtelomeric localization of mouse *Zscan4* gene at chromosome 7. (**B)** FACS analysis of global DNA methylation levels between WT G4 Terc$^{-/-}$ ES cells, and between control and 5-aza-dC treated ES cells. (**C**) Quantitative PCR shows relative gene expression levels of DNA methytransferases, *Tet* enzymes, and *Tbx3*. Error bars indicate mean ±SEM (n=2). **(D)** Bisulfate sequencing analysis of CpG dinucleotide of mouse *Tcstv1/3* promoter regions at -2.3 kb among WT, G4 Terc$^{-/-}$ and 5-aza-dC treated WT ES cells. *Tcstv1* and *Tcstv3* share the same promoter sequences. Open circles, unmethylated; closed circles, methylated. **(E)** Quantification of bisulfate genomic sequencing results of around 15 independent clones (n) at *Tcstv1/3* promoter**s**. Note the decrease in methylated CpGs at *Tcstv1/3* promoters in G4 Terc$^{-/-}$ compared with WT ES cells. Error bars indicate mean ± SEM. *, p<0.05; **, p<0.01, ***, p<0.001, compared to WT controls.

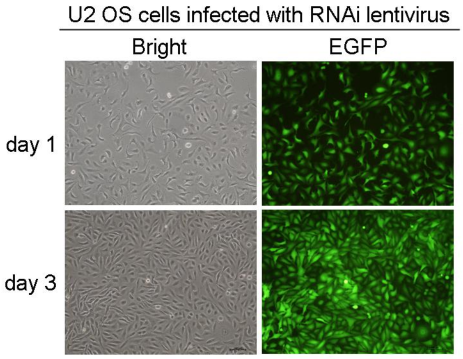

Figure S2. ALT U2 OS cells were infected with human ZSCAN4 RNAi lentivirus with high infection efficiency. Almost 100% of U2 OS cells were successfully infected with RNAi lentivirus at day 1 post-infection.

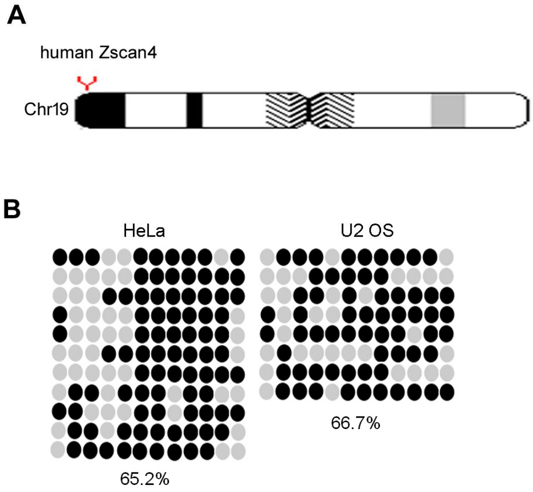

Figure S3. No noticeable difference in DNA methylation occupancy at human ZSCAN4 promoter between HeLa and U2 OS cells. **(A)** Subtelomeric localization of human ZSCAN4 gene at chromosome 19. **(B)** Quantification of bisulfate genomic sequencing results of 8-12 independent clones (n) at ZSCAN4 promoter. DNA methylation at ZSCAN4 promoter shows no noticeable difference between HeLa and U2 OS cells.

Table S1. 19 nuclotide sequences for RNA knockdown

Control shRNA	GTAGTTCGCCAGTTAATAG
hZscan4 shRNA4	GTAAAGTCTCTCTGAGAAT
hZscan4 shRNA5	GGGTCCATAAATGGAATCA
hZscan4 shRNA6	GGTCTTTAAGTATCTCTGT
hZscan4 shRNA7	GATACTTCCTTAGAAACAG
mZscan4 shRNA	GAGTGAATTGCTTTGTGTC

Table S2. Primers for qPCR analysis of telomere length

Gene name	Primer
mTel-F	CGGTTTGTTTGGGTTTGGGTTTGGGTTTGGGTTTGGGTT
mTel-R	GGCTTGCCTTACCCTTACCCTTACCCTTACCCTTACCCT
36B4-F	ACTGGTCTAGGACCCGAGAAG
36B4-R	TCAATGGTGCCTCTGGAGATT

Table S3. Primers used for real-time PCR analysis

Genes	Forward	Reverse
β-actin	GAGATTACTGCTCTGGCTCCTA	GGACTCATCGTACTCCTGCTTG
Zscan4	AAATGCCTTATGTCTGTTCCCTATG	TGTGGTAATTCCTCAGGTGACGAT
Tcstv1	TGAACCCTGATGCCTGCTAAGACT	AGATGGCTGCAAAGACACAACTGC
Tcstv3	AGAAAGGGCTGGAACTTGTGACCT	AAAGCTCTTTGAAGCCATGCCCAG
Gm4340	CGAGGCACTGGGTCTAAGAG	CCAATGAACAGGTCATGCTG
Dub1	GCAGGCCAACCTCAAACAG	CGCAGGGCTCTCCTAAATCTT
Eif1a-like	GGTAATGGAGTCCTGCTGTATTT	AGAGTCCTGGCTTCTGATAGT
Dnmt1	TGCAAGGCGTGCAAAGATATGGTG	TGGGTGATGGCATCTCTGACACAT
Dnmt3a	CAGCACCATTCCTGGTCATGCAAA	TCCTGTGTGGTAGGCACCTGAAA
Dnmt3b	GCACAACCAATGACTCTGCTGCTT	AGGACAAACAGCGGTCTTCCAGAT
Tet1	AGTTGCCTGCATGTATGTGTGTGC	GCAACAGCTGGCTAAACAACGGAA

Tet2	ATCGCCGGTAATGGCTTCTGATCT	AAGGTCTCCACTGACGCAAAGCTA
hβ-actin	AATGTGGCCGAGGACTTTGATTGC	AGGATGGCAAGGGACTTCCTGTAA
hZscan4	TACCCACCAGAGCAATGAGGGAAA	TGACACTCGGGACAAACAAATGGC
hTert	ATTCCTGCTCAAGCTGACTCGACA	ATGGTCTTGAAGTCTGAGGGCAGT
hTerc	TCTAACCCTAACTGAGAAGGGCGT	TGCTCTAGAATGAACGGTGGAAGG
MuERV-L	ATCTCCTGGCACCTGGTATG	AGAAGAAGGCATTTGCCAGA

Table S4. ChIP-qPCR primers of proximal regions of mouse *Zscan4c* promoter and human ZSCAN4 promoter

Primer	Forward	Reverse
Zscan4-pb1	GCATTATCTGTTCCTCTGGGTC	AACTCCTGTTCCTGGGTGGG
Zscan4-pb2	TGCTGGGTAAGGTAGTGTTT	CATCAGGCTAGGAGAAGTCA
Zscan4-pb3	TTCTTATCAATCCTTGCTCCCT	TCCTGCTCCAACATCTCCTC
Zscan4-pb4	TGGCAAACTCGTCTGAAGGT	CTTTGGGAAGGTCTTGGAAC
Zscan4-pb5	GGTAGGCGGGTAACACTCTG	GTGCTGCTGTGCTAGTGGAT
Zscan4-pb6	TGAAGACTGAGTTCGGAGGT	GAGAAGGAGGGATTGGAGCA
Subtel(chr7)	GCTGGACAGGTCAAGGTGGG	TGTAACGGCGAATGTGAGGG
Tcstv1/3 locus	TAAAGGCGTGAGCCACCACACTTA	TATGAGAGAGCAAGCAGGCCAAGT
mus β-actin	CGTGTGACAAAGCTAATGAGGCTG	CTAAGTTCAGTGTGCTGGGAGTCT
ZSCAN4-pb1	CCTGTATGAGGGTGGAAAGTG	AACATGAGGCTTGAGAGAGATG
ZSCAN4-pb2	ACTTCTCTGTGCCTCTGTTATTT	GACTCTGTTCTCAGTGCTTTCT
ZSCAN4-pb3	AGGTGGAGGTTGTGGTAAAC	CTTGGGCCTCGCAAAGATA
hu β-actin	TGCCTAGGTCACCCACTAATG	GTGGCCCGTGATGAAGGCTA
